# Supplementary material for: Pathophysiological Significance of Neutrophilic Transfer RNA-Derived Small RNAs in Asymptomatic Moyamoya Disease
Source: Cells. 2021 May 1;10(5):1086. doi: 10.3390/cells10051086 (PMC8147334; doi:10.3390/cells10051086)

| 2D Structure | Local AU | Position |
| --- | --- | --- |

```
154                                    183
                              8mer-1a
 5'-aaaAACTCTATGAGTGTAAGCAATGTGGGa-3' utr
       ||  :|||     |:  ||  :|  ||||||| 
 3'-uccUUAGGAUUGGCG-AUCUGGUACACCCu-5' tRF
                 1615 1413    7 6 5 4 3 2
               3' Pairing        Seed
```

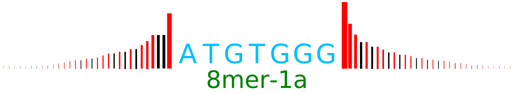


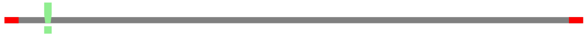

Supplement: Supplementary file 1 [file cells-10-01086-s001.zip › cells-1149161 3rd supplementary materials/Data Supplements/Data Supplement V.zip!/Data supplement 6-mRNAs targets of tsRNAs/tsRNA-Glu-TTC-010/Diagrams/tRF-Glu-TTC-010_vs_ENST00000304030(ZNF439).pdf]
